# “If You Are Not Circumcised, I Cannot Say Yes”: The Role of Women in Promoting the Uptake of Voluntary Medical Male Circumcision in Tanzania

**DOI:** 10.1371/journal.pone.0139009

**Published:** 2015-09-24

**Authors:** Haika Osaki, Gerry Mshana, Mwita Wambura, Jonathan Grund, Nyasule Neke, Evodius Kuringe, Marya Plotkin, Hally Mahler, Fern Terris-Prestholt, Helen Weiss, John Changalucha

**Affiliations:** 1 National Institute for Medical Research, Mwanza, Tanzania; 2 Centers for Disease Control and Prevention, Atlanta, Georgia, United States of America; 3 Jhpiego-an affiliate of John Hopkins University, Dar-es-Salaam, Tanzania; 4 MRC Tropical Epidemiology Group, London School of Hygiene and Tropical Medicine, London, United Kingdom; London School of Hygiene and Tropical Medicine, UNITED KINGDOM

## Abstract

Voluntary Medical Male Circumcision (VMMC) for HIV prevention in Tanzania was introduced by the Ministry of Health and Social Welfare in 2010 as part of the national HIV prevention strategy. A qualitative study was conducted prior to a cluster randomized trial which tested effective strategies to increase VMMC up take among men aged ≥20 years. During the formative qualitative study, we conducted in-depth interviews with circumcised males (n = 14), uncircumcised males (n = 16), and participatory group discussions (n = 20) with men and women aged 20–49 years in Njombe and Tabora regions of Tanzania. Participants reported that mothers and female partners have an important influence on men’s decisions to seek VMMC both directly by denying sex, and indirectly through discussion, advice and providing information on VMMC to uncircumcised partners and sons. Our findings suggest that in Tanzania and potentially other settings, an expanded role for women in VMMC communication strategies could increase adult male uptake of VMMC services.

## Introduction

Male circumcision provides partial protection against HIV infection acquired by men through vaginal sex by 60%-70%[1–4]. Tanzania is among fourteen sub-Saharan African countries with a high HIV prevalence and low male circumcision coverage that are scaling-up Voluntary Medical Male Circumcision (VMMC) for HIV prevention, as recommended by the World Health Organization (WHO) and the joint United Nations Programme on HIV/AIDS (UNAIDS)[5, 6]. Modeling exercises have estimated that scaling up VMMC to 80% coverage among males aged 15–49years will require 20.3 million circumcisions in these priority countries, and would result in 3.36 million new HIV infections averted and $16.5 billion in net savings by 2025[7, 8].

A national situation analysis conducted in 2009 in three regions in Tanzania showed high acceptability of male circumcision in both circumcising and non-circumcising communities: 76% of uncircumcised males indicated that they would get circumcised at health facilities if the services were available[9, 10]. The National AIDS Control Programme (NACP) of the Ministry of Health and Social Welfare (MOHSW) of Tanzania introduced the strategy for scaling-up VMMC in December 2010[11]. The strategy, which sets out a goal of circumcising 2.8 million men aged 10–34 years by 2015, focuses on 11 priority regions characterized by high HIV and low circumcision prevalence. The MOHSW specifically targets males in this age group to prevent new HIV infections by heterosexual sex among men. Currently, adult HIV prevalence in Tanzania is 5.1%, and HIV prevalence in Njombe and Tabora regions is 14.8% and 5.1% respectively. In a report from 2012, almost half of adult men in Njombe (49%) were circumcised; similarly in Tabora, circumcision prevalence is 56%[12].

In September 2009, the United States Agency for International Development (USAID) flagship Maternal and Child Health Integrated Program (MCHIP) implemented by the Johns Hopkins Program for International Education in Gynecology and Obstetrics (Jhpiego) together with the MOHSW, introduced VMMC services in Iringa region. The program has since grown to include three regions (Iringa, Njombe and Tabora), with more than 400,000 VMMCs provided as of September 2014 (Jhpiego database). However, among these, only 22% of clients were aged 20 years and above. The age distribution of clients raises questions about why few males aged 21–34 years came to be circumcised, and about the most effective strategies to increase the attendance of men over 20 years.

The MCHIP VMMC program has integrated aspects of community education into service delivery, including radio advertisements that describe benefits of VMMC, such as the prevention of some sexually transmitted infections (STIs), HIV, and cervical cancer prevention, as well as improved hygiene to educate communities surrounding the service about health benefits of VMMC. Some of the radio advertisements specifically target women by including female actors and highlighting women’s perspectives on VMMC. Radio shows have aired quarterly, addressing issues in VMMC including women’s involvement in promoting VMMC to their partners and sons. Separate brochures targeting female sexual partners and mothers have also been widely distributed in communities since 2011. Starting in 2013, female and male peer promoters have also reached out to men and women in communities where services are located with group and individual counseling and education on the benefits of VMMC. Women who accompany their sons or partners to the service are provided with information on benefits of VMMC, including the partial protection from HIV, and how to care for their sons or partners post-surgery, including discussion on the recommended six-week period of sexual abstinence.

The role of women in decision-making surrounding male circumcision uptake has been mostly described in the sub-Saharan African context[13–19]. In Zambia, a recent study on the predictors of men’s readiness to go for circumcision found that discussing VMMC with female partners was the strongest determinant of readiness for circumcision[20]. Also, studies in South Africa, Kenya and Tanzania show that women inform partners on the importance of VMMC and prefer circumcised men as partners[16, 18, 21, 22]. A study conducted in Kisumu, Kenya showed that 76% of the women preferred circumcised men as sexual partners[20]. Other studies have reported that pressure from women influences men to go for circumcision [16, 18, 21, 22]. In one study in Kenya, female partners reported that they have even ended relationships upon realizing that their partner was uncircumcised [18].

Women were often described as assuming a supportive role during the post circumcision period by caring for partners and reminding them to adhere to condom use even after being circumcised [15]. Further, some women may also go as far as scheduling appointments for partners to go for circumcision in health centers where it is available[19]. In a study conducted in Tanzania, both male and female community members agreed however, that women often used various ways to influence their partner’s decision in choosing to go for circumcision for example, by denying sex to uncircumcised partners [21].

This paper presents a detailed analysis of ways women influence uptake of VMMC as reported by men and women, from two regions in Tanzania.

## Methods

### Sampling and recruitment

In the formative research, two districts (one in each Tabora and Njombe regions) were purposively selected from a list of districts in the region that had received VMMC outreach services from MCHIP in the past twelve months. Outreach services were periodic VMMC services provided in selected health facilities not providing the standard static services provided by MCHIP. In each selected district, facilities were listed, stratified into rural and urban. Statistical software, Stata 12 (StataCorp, Texas, USA) was used to randomly select facilities from the list, to fill quotas for adequate number of facilities in each stratum. Two facilities were selected from each district, one from a rural area and another from an urban area. This qualitative study was part of formative research that preceded a cluster randomized controlled trial that evaluated an intervention to increase VMMC uptake in Tabora and Njombe regions in Tanzania.

Four circumcised individuals, who had been circumcised in the previous 7 weeks, aged 20–34 years per facility were randomly selected from the VMMC registers at the facility and contacted, either by telephone or via local elected leaders (street, village and sub village leaders) in the areas, to invite them to participate in in-depth interviews (IDIs). The majority of the leaders were males and had good rapport with young people in their areas. Uncircumcised men aged 20–34 years were suggested by informants and local leaders from the community in the catchment areas of the four selected facilities and contacted physically or via phone and invited to participate in the IDIs. Since the majority of local leaders were males, it was easier for them to identify uncircumcised males in their communities.

For the recruitment of the participatory group discussions (PGDs), the same local leaders helped recruit male and female participants from the community, and researchers screened and recruited participants through purposive sampling based on age, sex and residence. Circumcision status was not an eligibility criterion for male PGD participants.

### Data collection

Data were collected between February and March 2014. IDIs and PGDs were conducted in Swahili using semi-structured interview guides and were held in private locations such as in the participant’s home or nearby in quiet, secluded, outdoor areas. A total of 30 IDIs were conducted, with circumcised and uncircumcised men, and 20 PGDs were conducted among men aged 20–49 and women aged 20–34 years (Table 1).

**Table 1:** Sample of Health Facilities and Interview and Group Discussion Participants.

Region	Facility	PGDs (n = 20) (2 PGDs per age group)	In-depth Interviews (n = 30)
Njombe	Anglican Health Centre (urban) Ihalula Dispensary (rural)	Men 20–24 years Men 25–29 years Men 30–49 years Women 20–26 years Women 27–34 years	8 uncircumcised men 20–34 years 6 circumcised men 20–34 years
Tabora	Nata Health Centre (urban) Itobo Health Centre (rural)	Men 20–24 years Men 25–29 years Men 30–49 years Women 20–26 years Women 27–34 years	8 uncircumcised men 20–34 years 8 circumcised men 20–34 years

There were two age strata for women (20–26 years and 27–34 years) and three age strata for men (20–24, 25–29 and 30–49 years) (Table 1). The primary goal of the research was to explore the views of the males on the uptake of male circumcision although it was recognized that getting the views of women was important. Therefore there is a disparity of having more PGDs with males than with women.

On average, each PGD consisted of between 6 to 12 participants depending on the availability and willingness of participants to take part. All PGDs were conducted in single sex groups (by same sex researchers) to allow ease of conversation among participants. IDIs were conducted by male graduate researchers. All IDIs and PGDs were digitally recorded, and researchers wrote notes that were later expanded. At the end of each day, the field coordinator electronically sent all digital files to the data management unit at the National Institute for Medical Research in Mwanza for transcription. A senior social scientist listened to a proportion of the recorded interviews and PGDs (6 interviews and 4 PGDs) at the beginning of data collection in each district and provided feedback to interviewers and PGD facilitators as part of the quality check process. Recordings were transcribed into Swahili and then translated into English by certified translators

### Analysis

Data was analysed in stages. In the first stage, two researchers who were involved in data collection (HO and FM) independently listened to digital recordings and read interview notes immediately following completion of the IDIs and PGDs. Thereafter a data coding scheme was developed after consultation between two social scientists (GM and HO) and used for coding all data. Subsequently each of the two researchers (HO and FM) performed a thematic analysis by documenting the recurring themes from the findings. A third researcher (GM) compared these analyses to produce a richer discussion of the identified themes. The theme of *the role of women as a facilitator of VMMC* was prominent in the first two stages ofanalyses, so further thematic analyses were conducted to generate emerging sub-themes.

### Ethical oversight

The study was granted ethical approval by the National Research Ethics committee in Tanzania (reference number NIMR/HQ/R.8a/Vol.IX/1661) and the Ethics Committees of the London School of Hygiene and Tropical Medicine (reference number 6238–02) and the US Centers for Disease Control and Prevention (reference number 6474). All study participants provided either signed or thumbprint (for those could not read or write) consent.

## Findings

Overall, the majority of participants in group discussions mentioned an important role of women in men’s decision-making around seeking VMMC services: in PGD groups, it was discussed in 14 of the 20 groups (6 out of 8 of the women’s groups and 8 out of the 12 male groups). Mention of women as key people in their decision-making to seek VMMC in the IDIs with recently circumcised males was less prominent. Of the circumcised male IDI participants, approximately one third (5 of 14) mentioned the involvement of women in their decision-making process to get circumcised.

### Positive roles

#### Providing information about VMMC

Several PGD participants mentioned women as a source of information to their partners. One IDI participant explained that his wife was the one who brought up the possibility of him getting circumcised, although he reserved the decision-making for himself:

*She told me that*, *they have announced at her church that men who are not circumcised are supposed to go [get circumcised]…Because it is difficult for her to tell me*, *you must go*. *She cannot have more power to decide than me because she gave it as a thought*. *I took the thought into consideration and accepted it*, *that’s when I made the decision to go [for circumcision]*. (34, circumcised male, married, Njombe rural)

Male participants in PGDs also reported lacking information or ‘understanding’ on VMMC, and having women explain to them the benefits of VMMC. Male and female participants were in agreement that it is helpful to have women be knowledgeable on the benefits of circumcision.

*If you as a woman keep quiet*, *then your partner won’t know that he is in trouble*. *So*, *as a woman*, *you have to educate your partner and to persuade him by saying*, *my friend*, *you are supposed to go and get circumcised so that we can prevent ourselves from getting these sorts of diseases…such as this and that…in order for him to understand*. *I know that*, *if he truly loves you*, *he will understand and go for circumcision*. (Group discussion, 20–26 year old females, Tabora rural)

*For instance*, *if a man has married a woman who has been educated*, *that woman will advise her husband [on going for circumcision]…it is possible this a man’s wife has been well informed about the benefits of circumcision and that she understands that the male foreskin can possibly carry dirtiness which may increase the speed of disease infection*. (Group discussion, 20–24 year old males, Njombe rural)

In addition to providing information to male partners, a number of women in PGDs described assuming the role of an advisor to their partners concerning the importance of going for circumcision. Women, from both sites and in both rural and urban areas consistently reported that their influence on decision-making had to be indirect and careful.

*As a woman*, *you have to be your husband’s advisor*. *You have to tell him*, *this and that is not good…you have to go [for circumcision]*. *And you have to tell him gently*, *that is how he will understand…* (Group discussion, 27–34 year old females, Tabora urban)

In advising or persuading their male partners to go for circumcision, women explained that it was important to use ‘soft’ language to explain why circumcision was an important choice to make in order to get their point across.

*Women have a very high convincing power*. *You don’t have to fight about it that will ruin your marriage*. *You have to be gentle*, *and bring it up depending on his mood*. *You must use soft language and you have to use enough evidence when explaining why it is important for him to circumcise…such as if you have had a sexually transmitted disease…* (Group discussion, 27–34 year old females, Tabora rural)

Although a number of women were reported in being successful in advising their partners to go for VMMC, not all men who received advice from their partners proceeded to go for the procedure. Two IDI respondents (uncircumcised and married) from Tabora region, mentioned that they had received advice from their partners to go for VMMC but had made a personal decision to not go for it due their work responsibilities.

#### Pressuring partners to go for VMMC

Apart from the discussion around women providing information or advice, the denial of sex by female partners was prominently discussed among PGD and IDI respondents as a strong motivator for men to go for VMMC services. More than a quarter (4 out of 14) of the circumcised male respondents in IDIs reported being denied sexual intercourse by their partners, which prompted them to go for VMMC. Two participants were from Njombe (one rural and one urban) and two from Tabora region (one rural and one urban). One IDI respondent reported that circumcision was a condition for the fulfillment of his intimate relationship with his fiancée, which is what motivated him to go for VMMC:

*…One day when I met my partner*, *I had a guilty conscience*. *She told me*, *if you are circumcised then I will agree to be with you so that we can live together*. *But if you are not circumcised*, *I cannot say yes*. *At that*, *I had a guilty conscience and decided to go back [to get circumcised]…she is now my fiancée*. (24 years old, Circumcised male, unmarried, Njombe rural)

Another respondent described his experience of being sexually rejected due to his uncircumcised status. This refusal of sex was a strong facilitator to pursue VMMC.

*This girlfriend of mine*, *I seduced her on the street and went with her where I lived*. *As soon as I took off my clothes and she saw that I had that thing [foreskin]*, *she said no*. *She refused… Later on*, *when I heard that this service [VMMC] was available*, *that is when I decided to go*. *I went and got that service…I was pressured by her*. (24 years old, Circumcised male, unmarried, Njombe urban)

Participants of PGDs also discussed how women influenced men to go for VMMC through refusing to have sexual intercourse with uncircumcised men. Male and female groups were in agreement that the denial of sex by women acted as a form of social pressure to men to go for circumcision. A similar scenario was narrated by female PGD participants:

*A woman may get a male lover*, *now when they are together and he takes his clothes off*, *if she sees that he is uncircumcised since she has learnt [about circumcision] from the seminar…she will say*, *no…I cannot do it with you since you have not been circumcised*. *So*, *this will make him feel bad*, *he will think*, *why has she denied me while she loved me…*? *It must be because I am uncircumcised…So*, *that pressures him*. (Group discussion, 20–26year old females, Tabora rural)

Male and female participants, specified, however, that women were more likely to refuse sex to casual partners compared to husbands or long-term partners. Participants from rural areas also suggested that men are more likely to be denied sex when they are uncircumcised if they are in urban areas where there is a diversity of people and more men from other circumcising communities. Notably, the four IDI respondents who described being denied sex were unmarried however; experiences of men being denied sex were equally shared by men from rural and urban areas. Female PGD participants explained that married women have more difficulty suggesting or negotiating with their husbands to go for VMMC, as they have already engaged in long-term sexual relations despite their uncircumcised status. Because of this, a suggestion that the husband should get circumcised might elicit suspicion that the wife was engaged in extramarital affairs. One female participant said:

*You have to tell him indirectly*, *because if you just tell him I don’t want you because you are like this*, *he will misunderstand you*. *You don’t want me while all this time we have been together*, *today you tell me you don’t want me*? *…*.*You must have another man whom you’ve seen is not like this*. *So*, *for something like this*, *you have to take it slow*. *Because we are being educated like this*, *slowly*, *there is a day that he will agree*, *if he is your husband and he loves you*, *he will agree*. (Group discussion, 20–26year old females, Njombe rural)

#### Avoiding embarrassment from female sexual partners

Male participants also discussed going for circumcision to avoid feeling ashamed or embarrassed during sexual encounters, which was described as being extremely detrimental to confidence. One participant in a male group discussion explained:

*There are many reasons that make men go for circumcision*. *One is social pressure*, *if you try to have sex with any woman when you are uncircumcised*, *she will tell her friends*. *So*, *when she tells her friends*, *they will all laugh at you*. *You will have to go get circumcised*. (Group discussion, 20–24year old males, Njombe urban)

Embarrassment in revealing uncircumcised status was mostly mentioned in the context of sexual encounters, and more so among younger men compared to men in older age groups. However, PGD participants also mentioned men experiencing embarrassment from revealing their uncircumcised status to other men.

*Some men postpone going [for circumcision] because they don’t want to be found out that they are not yet circumcised*. *They feel embarrassed so*, *they wait for a chance to go alone…* (Group discussion, 20–24 year old males, Tabora urban)

#### Decision-making role as mothers

Although the majority of PGD participants identified fathers as the primary decision makers for their male children to get circumcised, mothers were still seen to have a very key role and generally very supportive of circumcision. Women from rural Njombe explained that, if a male child was uncircumcised, the mother is more likely to be the one to suggest the idea of circumcising the child, since the father might not think it important if he himself is not circumcised. Mothers often take the initiative of taking their boys to the health center for circumcision to protect them from embarrassment they might face in school for being uncircumcised. Two participants from group discussions elaborated on this:

*The mother is often the final decision maker on taking the child for circumcision that is if her husband has been circumcised too*. *If a man has already gone for circumcision*, *he will automatically understand the importance of taking the child to get circumcised too…* (Group discussion, 20–26 year old females, Njombe rural)

In addition to younger children, it was also mentioned that mothers can also influence their older sons to go for VMMC. One circumcised male IDI participant from Tabora who was influenced by his mother and intimate partner shared:

*I saw a lot of my peers who had come to get circumcised…and at home also*, *my mother insisted that I should go and get circumcised because circumcision is a good thing*. *She told me that*, *she had intended to take me to the Nzega hospital so that I would get circumcised*. *But now because there is a free service here*, *I should go*. *I refused at first…but then my girlfriend also told me the same thing again…and I started thinking about it…* (23 years old, Circumcised male, unmarried, Tabora urban)

### Non-supportive role

PGD participants also mentioned that sometimes, women, specifically married women, may not be supportive of VMMC and influence their male partners against going for circumcision. According to male and female PGD participants, this was based on a perception that if a man seeks circumcision, he may be looking for additional protection against HIV and other sexually transmitted infections so that he might engage in multiple concurrent partnerships more freely. One female participant explained:

*Also*, *some of us women prevent our husbands from going for circumcision*. *A woman would say*, *why are you going for circumcision while I loved you the way you are*? *Who has told you about this or do you have a mistress who has convinced you to get circumcised*? (Group discussion, 20–26 year old females, Tabora urban)

A married male participant also explained how some married women choose to go against their husbands’ decision or suggestion to go for VMMC.

*[Going for circumcision] becomes a problem because your wife is already used to you*, *you already have four children and all this time she sees you are uncircumcised*. *If you tell her you want to join your peers and go for circumcision she will be puzzled*. *She will say that she’s satisfied and she has been with you in that state [uncircumcised] and you have had children while you had the foreskin*, *now what are these things you are suggesting*? *Those are the kinds of questions we are asked…* (Group discussion, 30–49 year old males, Njombe rural)

## Discussion

A notable finding of this study was the widely held view among both male and female participants that women have an important role to play in an uncircumcised man’s decision of whether or not to get circumcised. Additionally, most participants reported widespread support among women for VMMC, although some women did report the tendency to become suspicious about infidelity and the rationale for pursuing VMMC. It was noted throughout that married women’s role in decision making is often indirect when it comes to advising or educating men about seeking circumcision. However, a powerful direct method that unmarried women use to influence a man’s decision is withholding sex or making circumcision a condition for establishing a sexual relationship. Our findings are similar to studies conducted in Iringa, Tanzania and Kisumu, Kenya where women also reported refusing to have sexual relationships with uncircumcised men and insisted that partners go for circumcision before they have sex [16, 18].

Women’s influence on decision-making appears to vary according to their marital status. Although a recent study in Iringa showed that both married and unmarried women report threatening to leave their partners if they refused circumcision[16], our findings are slightly different. Reports from participants in this study indicate that it is more difficult for married women to influence their husband’s decision to get circumcised. Married women are less likely to directly influence partners to take up circumcision by withholding sex as sex is considered a marital right. Studies around the dynamics of condom use reflect similar findings: partners in committed relationships have more difficulty negotiating condom use since it is perceived to entail infidelity and a lack of trust within the relationship[23–25]. Study participants also noted that married women may be less supportive of circumcision for the same reasons.

With this distinction between influences from married and unmarried women, VMMC programs that focus on women may consider engaging them during the mobilization and sensitization stage by structuring messaging differently for married and unmarried women. It is also evident that women do not act a homogenous group in their engagement in VMMC uptake. Additional research is needed to explore how best to engage women in different life circumstances in terms of age, marital status, and parenthood in VMMC programming.

While a few studies have reported how women influence men’s decision-making around VMMC[12, 13, 16–19, 21, 22], there is paucity of literature on the engagement of women in VMMC programs. Instances of integration of women and gender-focused communication into Kenya’s National Program of Voluntary Medical Male Circumcision under the Kenyan Ministry of Health and the National AIDS and STI Control Programme (NASCOP) have been presented, but evidence on outcomes of the integration is not described[26, 27]. Our findings and those from other studies [15, 16, 18] strongly suggest that the messages targeting women are having an effect in promoting VMMC in Tanzania and Kenya.

A study in Zimbabwe that explored the barriers and motivators to VMMC uptake among men of different ages reported high motivation to seek VMMC for non HIV-related factors such as improved hygiene, preventing cervical cancer in their female partner and increasing sexual pleasure[13, 22]. Further, women often refer to penile hygiene and increased sexual satisfaction as reasons for suggesting circumcision to their male partners [16, 18]. Our findings suggest that, recommending male circumcision to a committed partner often implies that there is a need to seek for protection from HIV and other STIs. This translates into suspicions and questions concerning fidelity within the relationship. Therefore there could be potential benefit of stressing non-HIV related benefits of VMMC, especially in the case of married couples.

VMMC programs may have lessons to learn from programs such as Prevention of Mother to Child Transmission (PMTCT) in the arena of involving partners who are not the target of the intervention [28, 29]. While clearly, the primary target of VMMC programs are males, our findings suggest that systematically and appropriately engaging women could substantially increase uptake of the service.

A limitation of our study is that the main objective was to broadly explore the facilitators and barriers of VMMC among adult men in the community, and thus, the research instruments were not exclusively dedicated to the role of women in decision-making to seek VMMC. However, the strong emergence of this theme through systematic analysis allowed us to further explore this topic and its prominent presence in participant responses reassured us that it is a strong and valid finding. Additionally, although our findings suggested high awareness of and support for VMMC by women, we did not measure their degree of understanding of VMMC service provision.

## Conclusions

It is clear that women have an important role to play in influencing uncircumcised men to seek circumcision in Tanzania and other similar settings, both in indirect ways such as suggesting and providing information, and in direct ways such as withholding sex. However, the factors allowing women to have direct influence are complex and vary according to social circumstances such as marital status. Further exploration of the role of women in effective VMMC program strategies is needed to enable program planners and policy makers to scale-up VMMC and achieve maximum HIV reduction in affected communities.
